# The Bacillus of Typhoid

**Published:** 1890-04-05

**Authors:** 


### HYGIENE.

### THE BACILLUS OF TYPHOID.

That enteric fever is frequently propagated by drinking water is certain, and no less so, as Pettenkofer has proved from the experience of Munich, that it is bred in the soil of localities, saturated with fæcal matters, and diffused by the ground air and emanations from the earth. But an interesting observation at Zitomir shows that it may be derived from infected buildings, and at the same time confirms the identification of the bacillus by Eberth and Gaffky. Though all the Russian troops stationed there drank the same water, and the telluric conditions of the several barracks were alike, those quartered in certain block suffered much more heavily than those in others. An examination of the dust in the former revealed vast numbers of bacilli, including an abundance of Eberth's, and a thorough purification of the barracks was followed by the cessation of the fever, all other conditicns of soil and water remaining as before, thus placing enteric so far in the same category as pneumonia, tuberculosis, and diphtheria.

